# Exosomes in skin photoaging: biological functions and therapeutic opportunity

**DOI:** 10.1186/s12964-023-01451-3

**Published:** 2024-01-12

**Authors:** Amirhossein Hajialiasgary Najafabadi, Mohammad Hasan Soheilifar, Nastaran Masoudi-Khoram

**Affiliations:** 1 Department of Quantitative and Computational Biology, Max Planck Institute for Multidisciplinary Sciences, 37077 Goettingen, Germany; 2 Department of Pathology, Research Group Translational Epigenetics, University of Goettingen, 37075 Goettingen, Germany; 3 Department of Medical Laser, Medical Laser Research Center, Yara Institute, ACECR, Tehran, Iran; 4 Endocrine Research Center, Institute of Endocrinology and Metabolism, Iran University of Medical Sciences, Tehran, Iran

**Keywords:** Skin photoaging, UV-induced signaling, Stem cell, Exosome

## Abstract

Exosomes are tiny extracellular vesicles secreted by most cell types, which are filled with proteins, lipids, and nucleic acids (non-coding RNAs, mRNA, DNA), can be released by donor cells to subsequently modulate the function of recipient cells. Skin photoaging is the premature aging of the skin structures over time due to repeated exposure to ultraviolet (UV) which is evidenced by dyspigmentation, telangiectasias, roughness, rhytides, elastosis, and precancerous changes. Exosomes are associated with aging-related processes including, oxidative stress, inflammation, and senescence. Anti-aging features of exosomes have been implicated in various in vitro and pre-clinical studies. Stem cell-derived exosomes can restore skin physiological function and regenerate or rejuvenate damaged skin tissue through various mechanisms such as decreased expression of matrix metalloproteinase (MMP), increased collagen and elastin production, and modulation of intracellular signaling pathways as well as, intercellular communication. All these evidences are promising for the therapeutic potential of exosomes in skin photoaging. This review aims to investigate the molecular mechanisms and the effects of exosomes in photoaging.

## Introduction

The harmful effects of ultraviolet (UV) irradiation on the skin, the largest organ in the body, have resulted in an increased demand for sun-damaged skin care products. Photoaging is the premature aging of human skin due to continuous exposure to UV radiation leads to significant alterations including, irregular pigmentation, telangiectasias, roughness, deep wrinkles, dryness, rhytides, elastosis, and precancerous lesions. Moreover, photoaged skin is associated with cellular and extracellular changes. These changes include high epidermal thickness, disorganization of collagen fibers, accumulation of dystrophic elastic fibers, cell genomic instability, as well as diminished viability, and morphological changes of keratinocytes and human dermal fibroblasts, all of which contribute to the pathogenesis of skin photodamage [1, 2].

Exosomes are nano-sized vesicles that serve as a subgroup of vesicles involved in cell-to-cell communication, containing bioactive ingredients such as lipids, proteins, and nucleic acids for cell-to-cell communications. Exosomes can be easily endocytosed and transfer their contents to recipient cells. Exosome therapy as a cell-free therapeutic intervention is correlated with lower risks of tumorigenicity and immunogenicity, reduced potential for uncontrolled cell differentiation and cell proliferation compared to stem cell therapy. Exosomes also show promise as vehicles for drug or gene delivery [3]. A large number of studies have demonstrated the therapeutic implications of stem cell-derived exosomes (including those derived from bone marrow mesenchymal stem cells, umbilical cord-derived mesenchymal stem cells, adipose-derived stem cells, and pluripotent stem cells) in age-related diseases, tissue regeneration, wound healing, and dermatological conditions [4]. The biological functions of exosomes have mostly been investigated in preclinical studies. For example, exosomal mmu-miR-291a-3p could exert anti- senescence effect in human dermal fibroblasts, through TGF-β receptor 2 signaling pathway and promote skin wound healing in aged mice [5]. Human umbilical cord blood-derived mesenchymal stem cells (UCB-MSCs)-derived exosomes which contain a high concentration of growth factors such as epithelial growth factor (EGF), have been found to increase collagen production and migration ability of normal fibroblasts. These stem cells-derived exosomes penetrate into the epidermis of skin samples in a time-dependent manner and increase collagen I and elastin while decreasing MMP1 expression [6]. Because of the similarity between the molecular mechanism of aging and photoaging, these findings hold promise for the potential use of exosomes in anti-photoaging-related cosmetics or therapeutics for skin rejuvenation and regeneration.. The cosmetic and therapeutic benefits of exosomes for skin care are mediated through their immunomodulatory function, reduction of oxidative stress, decreasing senescence, and stimulation of extracellular matrix (ECM) components production. The aim of this review is to provide an overview of the molecular mechanism of UV-induced skin aging and to highlight the efficiency of exosomes in skin photoaging.

## Photoaging molecular mechanisms and related pathways

Photoaging is one of the most common skin defects. In the recent years, many studies have been conducted to understand the underlying mechanisms of skin aging. It has been discovered that a multitude of signaling pathways and molecules are involved in regulating this process [7]. In the subsequent section, we will provide an overview of the current understanding of the mechanisms involved in photoaging.

### Inflammation-related pathways

Many inflammatory pathways activated in response to UV radiation contribute to the generation of reactive oxygen species (ROS) and the degradation of collagen and elastin, which are two proteins responsible for skin elasticity and firmness. Interleukin-1 alpha (IL-1α) and interleukin-1 beta (IL-1β) are proinflammatory cytokines that are suggested to play a role in the photoaging process. In response to UV radiation, these cytokines are produced and contribute to the inflammation and damage caused by ROS. IL-1α and IL-1β can facilitate the breakdown of collagen and elastin by upregulating the expression of matrix metalloproteinases (MMPs), enzymes responsible for the degradation of these proteins [8]. Similarly, cytokine, like IL-6, can contribute to the breakdown of collagen and elastin by increasing the expression of MMPs. Additionally, IL-6 can promote the formation of senescent cells, which are damaged cells that have stopped dividing and can contribute to the aging process [9]. In addition, Toll-like receptors (TLRs), a type of receptor found in the body's immune system [10] are triggered by UV radiation, resulting in a cascade of inflammatory responses in the skin and finally leading to signs of aging [8]. TLR4 signaling pathway may contribute to the increased amount of IL-6 and IL-8 in the senescent skin cells following UV exposure [11]. UV radiation can induce expression of COX-2, which can lead to inflammation and skin damage in the context of photoaging. UV light-induced MAPK pathway can eventually promote COX-2 production [12, 13]. Other pro-inflammatory cytokines, such as TNF-α and IL-1β, can also enhance COX-2 synthesis [14]. Moreover, a recent study argued suppression of COX-2 can decrease the UV-induced consequences, underscoring the importance of this protein in photoaging [15].

### Oxidative stress-related pathways

UV radiation causes the production of ROS in skin cells, leading to oxidative stress. This stress causes damage tocellular components such as lipids, proteins, and DNA, which can lead to cellular dysfunction and ultimately contribute to the signs of photoaging, such as wrinkles, age spots, and loss of skin elasticity. The Nrf2/ARE pathway is a key regulator of the cellular response to oxidative stress, and it plays an important role in protecting skin cells from the damaging effects of UV radiation in photoaging. Under normal conditions, NF-E2-related factor-2 (Nrf2) is sequestered in the cytoplasm by its inhibitor protein, Keap1. However, in response to oxidative stress, Nrf2 dissociates from Keap1 and translocates to the nucleus, where it binds to the antioxidant response element (ARE) in the promoter region of genes that encode antioxidant and detoxification enzymes [16–18]. This leads to the activation of these genes and the subsequent synthesis of antioxidant and detoxification enzymes, that help neutralize ROS and prevent oxidative damage [19]. It was shown that upregulation of antioxidant enzymes' expression levels in human skin fibroblasts (HSF) via modulation of the KEAP1-Nrf2/ARE signaling pathway enhances cell antioxidant capacity and reduces UVA-induced ROS and lipid oxidation product malondialdehyde (MDA) [20]. Peroxisomes and peroxisomal enzymes also play a crucial role in regulating the levels of ROS. Investigators indicated the efficiency of catalase and superoxide dismutase in photoaging progression collapses significantly [21].

### DNA damage and repair pathways

UV can cause various types of DNA damage, including the formation of pyrimidine dimers (such as thymine dimers), which distort the DNA structure and interfere with normal replication and transcription processes. Moreover, it can lead to the generation of reactive oxygen species and indirectly cause nuclear DNA damage. Base-excision repair is responsible for repairing this type of damage, while UVB radiation directly damages DNA and is repaired through nucleotide excision repair [22]. As individuals age, the efficiency of various DNA repair mechanisms, including NER, BER, double-strand break repair, and mismatch repair, declines [23]. This results in a gradual accumulation of DNA damage over time, particularly in intrinsic aging, which can give rise to aging-related traits. UV exposure can exacerbate this process by causing more DNA damage. Concerning photoaging, prolonged exposure to UV radiation can lead to the accumulation of photoproducts in the skin, surpassing its DNA repair capacity [24]. Moreover, evidence suggests that UV-induced telomere mutations, shortening, and telomerase dysfunction might facilitate photoaging and cell death progression [23, 25].

In photoaging, the accumulation of DNA damage can trigger the persistent activation of the p53 pathway, which can contribute to the loss of skin elasticity and the development of wrinkles. Additionally, the ATM/ATR pathway is involved in the response to DNA damage. It activates DNA repair mechanisms and can induce cell cycle arrest to facilitate DNA repair. These pathways can also induce apoptosis if the damage is too severe or if the repair mechanisms are overwhelmed [26, 27]. Poly (ADP-ribose) polymerase-1 (PARP-1) is a well-studied nuclear enzyme that belongs to the PARP superfamily. PARP-1 functions as a sensor for DNA damage.. Upon detecting DNA damage, PARP-1 utilizes NAD+ as a substrate to add mono-ADP-ribose or poly (ADP-ribose) (PAR) to various acceptor proteins, including PARP-1 itself. Subsequently, activated PARP-1 can induce DNA repair through a base excision repair [28, 29]. However, high UV exposure can also lead to excessive activation of PARP-1 and therefore lead to depletion of the cellular stores of NAD+ and ATP, which can contribute to cell death [30].

### Apoptosis pathways

One of the important mechanisms implicated in the photoaging of the skin tissue is programmed cell death or apoptosis. It has been shown that there are a vast number of mechanisms underlying this process during photoaging, and many of them still remain unclear. The cascade begins with the dysregulation of crucial apoptosis-related proteins, including Bax, Bcl-xL, PARP, and caspases. [31]. One study discovered that induced deregulation in apoptotic genes, such as p53, caspase-8 and 3, Bax, and Bcl-2 can interestingly enhance anti-photoaging effects by preventing UVB-induced apoptosis [32]. Furthermore, UV might induce upregulation of MAPK pathway-related genes in the chemokine signaling pathway—resulting in oxidative stress and necrotic cell death [33]. On the other hand, it is shown that UV exposure can directly and indirectly (via induced ROS production) activate the mechanism of neutrophil extracellular traps (NET or netosis) which is an immune programmed cell death pathway in that neutrophils release their DNA and sacrifice themselves. Therefore, UV-induced netosis is suggested as a novel pathway that contributes to photoaging progression [34].

### Extracellular matrix (ECM) degradation-related pathways

Extracellular matrix (ECM) degradation is one of the main hallmarks of photoaging. Exposure to UV radiation can cause damage to the ECM by inducing the production of MMPs, which are enzymes responsible for breaking down collagen and elastin [35]. The stimulation of the MAPK pathway is the primary regulator of UVR-induced MMP upregulation. In addition, ROS generation is essential for UVR-induced MAPK-mediated signal transduction [36]. The UV-dependent MAPK induction results in MMP-1 overexpression followed by type I collagen (COL-1) degradation [37]. Moreover, another study suggested that inhibition of ERK and p38 protects against UVB-induced photoaging by promoting COL-1 accumulation [38].

In the skin, TGF signaling inhibits keratinocyte development and acts as a profibrotic agent in the dermis. In photoaging, chronic UV exposure triggers the TGF1/SMAD3 signaling pathway and leads to metalloproteinase-induced collagen breakdown and photo inflammation. UV irradiation also induces gene alterations in TGF pathway components such as TGFRI, TGFRII, SMAD2, and SMAD4 [39]. Furthermore, several studies support the idea that increased pro-collagen production through TGF-β/Smad pathways, and the expression suppression of MMPs by blocking MAPKs, AP-1, and NF-κB pathways could exhibit anti-photoaging effects [40–43].

### Autophagy-related pathways

Autophagy is a cellular process that involves the degradation and recycling of damaged or dysfunctional cellular components [44]. In the context of photoaging, studies showed that UV exposure can both induce and inhibit autophagy in a context-dependent manner.. Autophagy plays a complex role, with both protective and harmful effects [45, 46]. On one hand, autophagy can help to remove damaged proteins and organelles and can promote cell survival in response to oxidative stress and DNA damage caused by UV radiation. Autophagy can also help to maintain cellular energy homeostasis, which can be disrupted in response to UV radiation [47]. Specifically, exposure to UVB radiation leads to the direct and rapid activation of three proteins including AMPK, UVRAG, and p53, which in turn activate autophagy [45, 48, 49].

Autophagy can be inhibited by UV radiation and subsequent pro-inflammatory signals such as TNF-α, IL-1β, and IL-6 [50]. This inhibition of autophagy can contribute to the accumulation of damaged proteins and organelles, leading tocellular dysfunction and development of photoaging [51].

Chronic exposure to UVA irradiation decreases the expression of Bach2 (BTB and CNC homology 1, basic leucine zipper transcription factor 2) in skin fibroblasts,which increases the expression of cell senescence-related genes and enhances UVA-induced photoaging. Conversely, overexpression of Bach2 can decrease the expression of cell senescence-related genes. Bach2 plays a critical role in suppressing UVA-induced cell senescence via autophagy by modulating the expression of autophagy-related genes and directly interacting with autophagy-related proteins. The precise molecular mechanism underlying the connection between Bach2 and autophagy remains unknown, and further studies are necessary to elucidate this signaling pathway [52]. Also, another more recent study revealed that autophagy inhibition can result in higher photodamage in fibroblasts. It was shown that colony-stimulating factor 2 (CSF2) can enhance autophagy while decreasing the expression level of MMP-1 and MMP-3. The negative correlation between autophagy and mentioned MMPs supports the importance of autophagy in anti-photoaging response. Moreover, the expression of AKT can influence the activation of autophagy, which is overexpressed along with the JAK2/STAT3 pathway and may contribute to several severe UV-induced consequences [46]. Collectively, the impact of autophagy during photoaging depends on its balance with apoptosis induction, while more studies are needed to investigate the impact of autophagy in photoaging.

### Stress response pathways

Heat shock protein 27 (HSP27), a member of heat shock protein family, has been implicated in various cellular processes, including stress response, apoptosis, and cytoskeletal organization [53]. HSP27 has been shown to interact with several proteins involved in the regulation of oxidative stress, apoptosis, and aging, such as Bcl-2, p53, p21, and p16 after UV exposure [54]. Reduction in HSP27 expression has been associated with increased levels of MMP-1 and MMP-3, along with the downregulation of type I collagen [55]. Furthermore, the suppression of HSP27 expression can partially enhance apoptosis through further activation of p65 and caspase-3 [56]. These interactions can modulate the balance between cell survival and death, ECM degradation, and oxidative stress response in response to UV radiation.

### Skin Adipose tissue collapse

Skin-associated adipose tissue, consisting of dermal (DWAT) and subcutaneous (SWAT) adipocytes, is critical in skin photoaging. In particular, DWAT, located in the reticular dermis of the skin, serves as a unique layer of adipocytes that can extend into the upper dermis and create a "fat bridge" between the skin surface and subcutaneous fat, linking the area directly exposed to UV radiation with the deeper fat layer [57, 58]. However, the turnover rate of DWAT adipocytes exceeds that of SWAT, and long-term excessive exposure to UV radiation can lead to DWAT depletion and skin fibrosis due to adipocyte-myofibroblast transition [59, 60]. This transition results in the replacement of fibrosis with DWAT volume, causing an uneven skin structure and the formation of skin folds [61]. UV radiation induces the activation of the TGF-β signaling pathway, which contributes to the conversion of adipocytes to myofibroblasts, resulting in the depletion of DWAT [62].

In addition to DWAT, SWAT also plays a crucial role in skin photoaging [63, 64]. Proinflammatory chemokines (IL-6 and IL-8) deregulation and their regulatory pathways (JAK pathway) due to UV-induction can lead to SWAT depletion and thinning of connective tissue, resulting in skin atrophy and wrinkle formation [65]. Moreover, chronic UV radiation inhibits the differentiation of preadipocytes and reduces the accumulation of triglycerides in mature adipocytes due to the decrease in lipid synthesis, including acetyl-CoA carboxylase (ACC), fatty acid synthase (FAS), stearoyl-CoA desaturase (SCD), sterol regulatory element binding proteins (SREBPs), and peroxisome proliferator-activated receptors (PPARγ) expression [66]. The decrease in both DWAT and SWAT contributes to the overall deterioration of skin structure and function in photoaging.

## Exosome biogenesis, secretion, uptake, and function

**Fig. 1:**
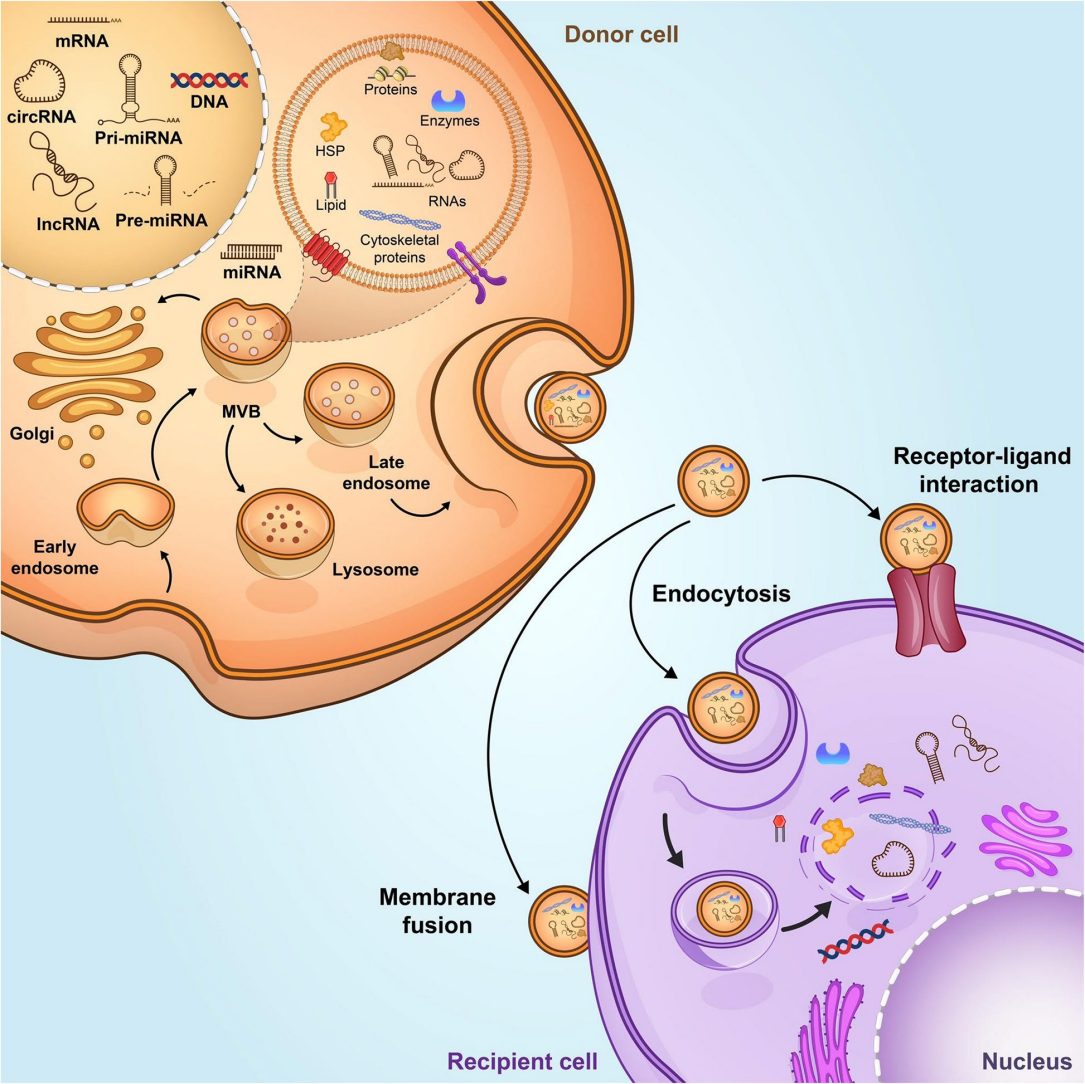
Exosome are small membrane vesicles that are formed by internalization of plasma membrane and formation of early endosomes. The early endosomes transform to late endosomes through maturation, then late endosomes, which termed as multivesicular bodies (MVBs), undergo inward membrane budding intraluminal vesicles (ILVs). MVBs fusion with the plasma membrane leads to release ILVs, or exosomes, into the extracellular space. Exosomes contain various biomolecules depends on the cell type of origin. Lipids, proteins and nucleic acids are the common molecular constituents of the majority of exosomes [67]. Exosomes are also rich in cytokines, growth factor and antioxidant

Exosomes are a subclass of extracellular vesicles with a size less than < 150 nm in diameter that facilitates intercellular communication [67]. Exosome biogenesis begins with formation of early endosomes through the invagination of plasma membrane which later generates multivesicular bodies (MVBs) containing Intraluminal Vesicles (ILV) (Fig. 1). During maturation of early endosomes to late endosomes or MVBs, the cargoes are incorporated into ILVs. ILVs are formed through the (endosomal sorting complex required for transport) ESCRT-regulated mechanism. The ESCRT is a family of proteins consist of ESCRT-0, -I, -II, -III, and Vps4 which are essential for vesicle budding, cargo sorting, and the formation of ILVs [68]. Recent evidence showed there is a second mechanism for exosome formation and cargo sorting in an ESCRT-independent manner which involves proteins such as tetraspanin [69]. The MVBs can fuse with the plasma membrane to release ILVs, which are called exosomes, to the extracellular environment. Exosomes include various proteins that participate in the formation and secretion of vesicles (Rab GTPase), proteins, major histocompatibility complex (MHC) proteins (MHC I and MHC II), tetraspanin family, heat shock proteins, and cytoskeleton proteins. Exosomes may carry other cell-specific proteins which their presence depends on pathophysiological conditions [68, 70].

The release of exosome is regulated by the SNARE proteins, RABs, and other Ras GTPase proteins. Rab GTPases is the member of Ras superfamily of GTPases and is responsible for the formation, membrane fusion, and secretion of vesicles. There are four Rab GTPase proteins including RAB7, RAB11, RAB27, and RAB35, which are involved in the formation and release of exosome. SNARE proteins mediate the fusion exosome with the plasma membrane or the membrane of organelles [71]. After fusion with plasma membrane, exosomes are released into the extracellular environment and deliver signals to recipient cells through different mechanisms. They can directly merge with the cell membrane and release their contents, interact with cell surface receptors through exosomal surface proteins, or undergoes endocytic uptake [70].

Cells can release exosomes with different sizes, contents, and functional effects on the target cells. At present, different methods are used to separate distinct subpopulations of exosomes. Among them, ultracentrifugation is the most common method that can separate exosomes based on their size and density. Other methods such as polymer precipitation, size-exclusion chromatography, and immunoaffinity are also used to isolate exosomes [72]. The isolated exosomes are then characterized by analyzing the exosomal markers. Exosomes contain two types of protein. The first group is the common proteins including tetraspanin family (CD9, CD63, CD81), cytoskeletal proteins (actin, tubulin), heat shock proteins (HSP70, HSP90), and the presence of exosome can be confirmed by identification of these proteins. Other specific proteins are varying depending on the cell of origin, for example exosomes derived from malignant tumors contain tumor antigens, which can be used to determine the origin of exosome, related disease and response to the specific treatment [73]. Besides proteins, exosomes contain lipids, mRNA, and other small RNA such as miRNA and other non-coding RNAs. Exosomes have the ability to transfer their genetic contents into the recipient cells and modify different cellular functions. Moreover, they have the potential to be used as diagnostic biomarkers or therapeutic tools for different pathologies [67].

## Stem cell-derived exosomes in photoaging

Some studies have indicated that different cells including stem cells and non-stem cells can release exosomes and exert therapeutic effect against photoaging (Table 1), which will be discussed in the next sections.

**Table 1:** In vitro and in vivo studies have proved the therapeutic potential of exosomes in amelioration of skin photoaging

Exosome source	Cargo	Signaling pathway	Study model	Ref
HucMSC	14–3-3ζ	SIRT1 pathway	HaCaT cells, Rat model of acute photodamage	[74]
ADSCs	miR-1246, lncRNA H19	Nrf2, MAPK/AP-1, TGF-β/Smad	HSF cells, Kunming mice UVB-irradiated mice	[75, 76]
BM-MSCs	miR-29b-3p	MAPK/AP-1	HDF cells	[77]
iPSCs	N/A	TGF-β	HDF cells	[78]
HDF	miRNA-22-5p, miR-133a, miR-223	MAPK, TGF-β/Smad	HDF monolayer and spheroids, nude mouse model	[79, 80]
HUVEC	N/A	Cell proliferation, collagen synthesis	HDF	[81]
Fungi extract	miR-CM1	Mical2-inuced aging	HaCaT, Kunming mice	[82]

### Human umbilical cord mesenchymal stem cells

Human umbilical cord mesenchymal stem cells (HucMSCs) are mesenchymal stem cells that are collected from the different parts of the human umbilical cord. These cells possess the ability to self-renew and differentiate into multiple cell types, including osteoblasts, chondrocytes, and adipocytes. HucMSCs exhibit immunomodulatory, anti-inflammatory, and anti-oxidative properties, making them promising candidates for cell therapy and regenerative medicine [83].

Recent studies have investigated the effects of HucMSC-derived exosomes on mitigating the harmful consequences of UV exposure on the skin. Specifically, researchers focused on the role of 14–3-3ζ, a protein found in HucMSC exosomes, and its interaction with SIRT1. The study demonstrated that HucMSC exosomes containing 14–3-3ζ could effectively protect skin cells from UV-induced damage by reducing oxidative stress and inflammation by mediating the SIRT1 pathway [74]. Moreover, these exosomes can enhance the proliferation and migration of HaCaT keratinocytes while inhibiting UVB-induced damage. The findings also show that these exosomes can reduce apoptosis and senescence, increase collagen type I expression, and decrease matrix metalloproteinase (MMP1) expression in photo-aged skin cells [84, 85].

### Adipose tissue-derived stem cells

The process of adipocyte development from mesenchymal cells is a multifaceted series of events, both transcriptional and non-transcriptional, that takes place throughout the lifespan of humans. Cells with preadipocyte traits can be derived from adipose tissue in adult individuals and can be grown in vitro. These cells can then be encouraged to differentiate into adipocytes [86].

The role of exosomes derived from adipose tissue-derived stem cells (ADSCs) in preventing photoaging has been extensively studied. Studies indicate that these exosomes effectively inhibit UVB-induced cellular DNA damage through ROS downregulation. Moreover, they can also significantly prevent MMP-1, MMP-3, and COL-3 overexpression and, therefore, protect the ECM integrity. These exosomes may also regulate Nrf2 and MAPK/AP-1 and activate TGF-β/Smad pathways upstream of the latter ones [87, 88].

Furthermore, studies have also shown that miR-1246, a highly prevalent nucleic acid in ADSC-derived exosomes, inhibits the MAPK/AP-1 signaling pathway to reduce MMP-1 production and activates the TGF-β/Smad pathway, resulting in enhanced pro-collagen type I secretion and an anti-inflammatory impact. In-vivo experiments on Kunming mice demonstrated that miR-1246 might protect against UVB-induced skin photoaging by inhibiting the production of wrinkles, epidermal thickening, and collagen fiber loss. Together, these findings suggest that exosomes derived from ADSCs, particularly miR-1246, play a vital role in the treatment of photoaging by regulating various signaling pathways [75]. Moreover, lncRNA H19, a reach component of ADSC-derived exosomes, shows MMP inhibition and COL-1 production effect on UVB-irradiated mice. It can also sponge miR-138 to target SIRT1, therefore mediating SIRT1 expression and its anti-photoaging impact [76].

### Bone marrow mesenchymal stem cells

Bone marrow mesenchymal stem cells (BM-MSCs) are a type of adult stem cells that have great therapeutic potential in regenerative medicine. Exosomes secreted by BM-MSCs have emerged as a crucial component of their paracrine signaling mechanisms. BM-MSC-derived exosomes contain a variety of bioactive molecules, such as growth factors, cytokines, and miRNAs, that can promote tissue repair and regeneration in various injury and disease models [89].

It is shown that BM-MSCs can mitigate UV-induced oxidative stress and inflammation in a dose-dependent manner and increase cell viability in human dermal fibroblasts (HDFs). BMSCs-exosomes also reduced the expression of MMP-1 and MMP-3 while promoting the expression of COL-1 by reversing MAPK/AP-1 pathway [90]. Moreover, miR-29b-3p, which is found in BM-MSCs-derived exosomes, can participate in reversion of UVB-induced HDF migration suppression, oxidative stress increase, and apoptosis promotion. It is suggested that mentioned miRNA can target MMP-2 and thus prevent COL-1 degradation [77].

### Induced Pluripotent stem cells

Induced pluripotent stem cells (iPSCs) are a type of stem cells that are generated by reprogramming adult cells, such as skin cells, to an embryonic-like state. iPSCs have the ability to differentiate into virtually any cell type in the body and have significant potential for regenerative medicine and drug discovery. iPSCs were first successfully created in 2006 by reprogramming human skin cells using a combination of four transcription factors, including Oct4, Sox2, Klf4, and c-Myc. This discovery was a significant breakthrough in the field of stem cell research and has led to a greater understanding of cellular reprogramming and its potential applications in the future [91–93].

It was observed that exosomes derived from human iPSCs (iPSCs-Exo) promoted the proliferation and migration of HDFs under normal conditions. Upon UVB irradiation, HDFs were damaged and overexpressed matrix-degrading enzymes (MMP-1/3), but pretreatment with iPSCs-Exo inhibited these damages. iPSCs-Exo also increased the expression of collagen type I in photo-aged HDFs. Furthermore, iPSCs-Exo significantly reduced the expression of SA-β-Gal and MMP-1/3 and restored the expression of COL-1 senescent HDFs [78]. SA-β-Gal is known to be a switch that shifts cells toward senescence fate and is known as an aging marker [94]. Therefore, these results suggest that iPSCs-Exo may have therapeutic potential in the treatment of skin aging.

## Exosomes-derived from other cells

Human dermal fibroblasts (HDFs) are the main cells in skin derived from MSCs, which play a critical role in extracellular matrix (ECM) remodeling and providing integrity and elasticity to the skin. In the process of skin aging, HDFs proliferation is declined, with decreased collagen production and increased MMPs, resulting in the degradation of the ECM. All of these processes lead to loss of integrity and elasticity and the formation of wrinkles.

Exosomes secreted by human dermal fibroblast cell UVB-irradiated human dermal fibroblasts (UVB-HDFs) are associated with skin photoaging. The analysis of miRNA expression profiling showed the number of dysregulated miRNAs in extracellular vesicles (EVs) derived from UVB-irradiated HDF. Upon UVB-irradiation, expression of miRNA-22-5p was significantly increased in HDF cells and their derived EVs, and can be transferred to other HDFs cells. further analysis showed that miRNA-22-5p upregulation promotes photoaging by targeting growth differentiation factor 11 (GDF11), a protein that protects HDF cells from photoaging [79]. In another study, exosomes derived from three-dimensional (3D) aggregation of HDF cells or spheroid induced collagen synthesis and reduced inflammation in a photoaged skin of mice model. It was hypothesis that miR-133a and miR-223 were upregulated and miR-196a was downregulated in the exosome derived from 3D cultured HDF spheroids, which might inhibit MMP expression, enhance collagen restoring and replacing and activate TGF-β signal pathway. Thus 3D HDF-XOs can be used as an effective approach to prevent skin photoaging [80].

Human umbilical vein endothelial cell (HUVEC) is a model cell line to study endothelial cells and can be derived from umbilical cords. Recently, Ellistasari et al. have conducted an in vitro study to investigate the effect of exosomes derived from HUVEC cells in attenuating skin photoaging. They observed that Exo-HUVEC can markedly increase cell proliferation and collagen synthesis in UVB-irradiated fibroblasts, Moreover, Exo-HUVEC can decrease MMP expression which leads to inhibiting collagen degradation in the photoaged cell line model. This source of exosome has the potential efficiency to prevent and treat skin photoaging [81]. Exosome sources are not limited to animal cells. Interestingly, natural exosomes, that originate from plants or other organisms, contain more bioactive molecules than those derived from animal cells. In the study by Han et al. exosome-like nanovesicles derived from a medicinal mushroom, Phellinus linteus (PL), has been shown to have anti-aging and anticancer effects. The fungi exosome-like nanovesicles (FELNVs) can protect skin from UV-induced photoaging. It was shown that fungal EVs are enriched with different miRNAs including miR-CM1-5, and among them miR-CM1 could protect HaCaT cells from UV-induced damage. MiR-CM1 exerts a protective effect through reduction of aging-related markers such as SA-β-Gal, ROS level, MMP1, and COL1A2 expression. Mical2 was known as a direct target of miR-CM1 which is involved in the regulation of age-related processes [82].

## Clinical applicability of exosomes in photoaging: current status and perspectives

In recent years, exosomes have been exploited as a novel candidate for treatment of many diseases including central nervous system disorders, cardiovascular diseases, and cancer. Under the pathophysiological condition, biological components of exosomes are changed, reflecting the alteration in the cell functions. The alteration in the exosomal components can be served as diagnostic and prognostic biomarkers in many diseases from cancer to aging [95]. Exosomes can be extracted from cell culture, tissues, and biological fluids including plasma, serum, urine, etc. [96]. Exosomes can act locally or transported to distant tissues via body fluids and modulate the function of target cells [97].

Mesenchymal stem cells are multipotent stem cells that that possess a the high ability to release exosome and can be extracted from bone marrow, umbilical cord, and adipose tissue [98]. Exosome therapy as a cell-free strategy offers severaladvantages of small size, no risk of tumorigenicity, and long-term storage making it a potentially safer and more effective alternative to stem cell therapy [3]. Also, exosomes show great promises as the drug delivery carrier due to high stability, biocompatibility, and low immunogenicity compared to virus-based delivery and other non-viral methods. However, there are still some challenges for the application of exosomes in clinics such as low yield of isolation [72].

Preclinical investigations showed that exosomes may have a therapeutic role in aging and other age-related diseases [99]. Cellular aging is due to various biological changes including, epigenetic alteration, genomic instability, senescence, oxidative stress, mitochondrial decline, and dysregulation of intracellular communication [100]. Some studies have demonstrated the therapeutic potential of exosome in preclinical models of age-related diseases such as Alzheimer’s, Type 2 diabetes (T2DM), osteoarthritis, chronic kidney disease, etc. [99].

Exosomes have many beneficial effects for skin care as they contain various biological molecules that can help to promote skin repair and regeneration [101]. Previous studies have demonstrated that exosomes and other EVs have therapeutic benefits in skin defects such as wound and aging. Most of these studies on the potential use of exosomes in skin repair have been conducted in animal models. For example, it was found that bioengineered exosomes loaded with miRNA-542-3p, derived from bone marrow MSCs (BMMSCs), could promote cell proliferation, collagen synthesis, and wound closure in mice models. Currently, the clinical applicability of exosome-based therapy is limited to skin wound repair [102]. To date, there is no clinical trial has been conducted on exosome in photoaging.

**Fig. 2:**
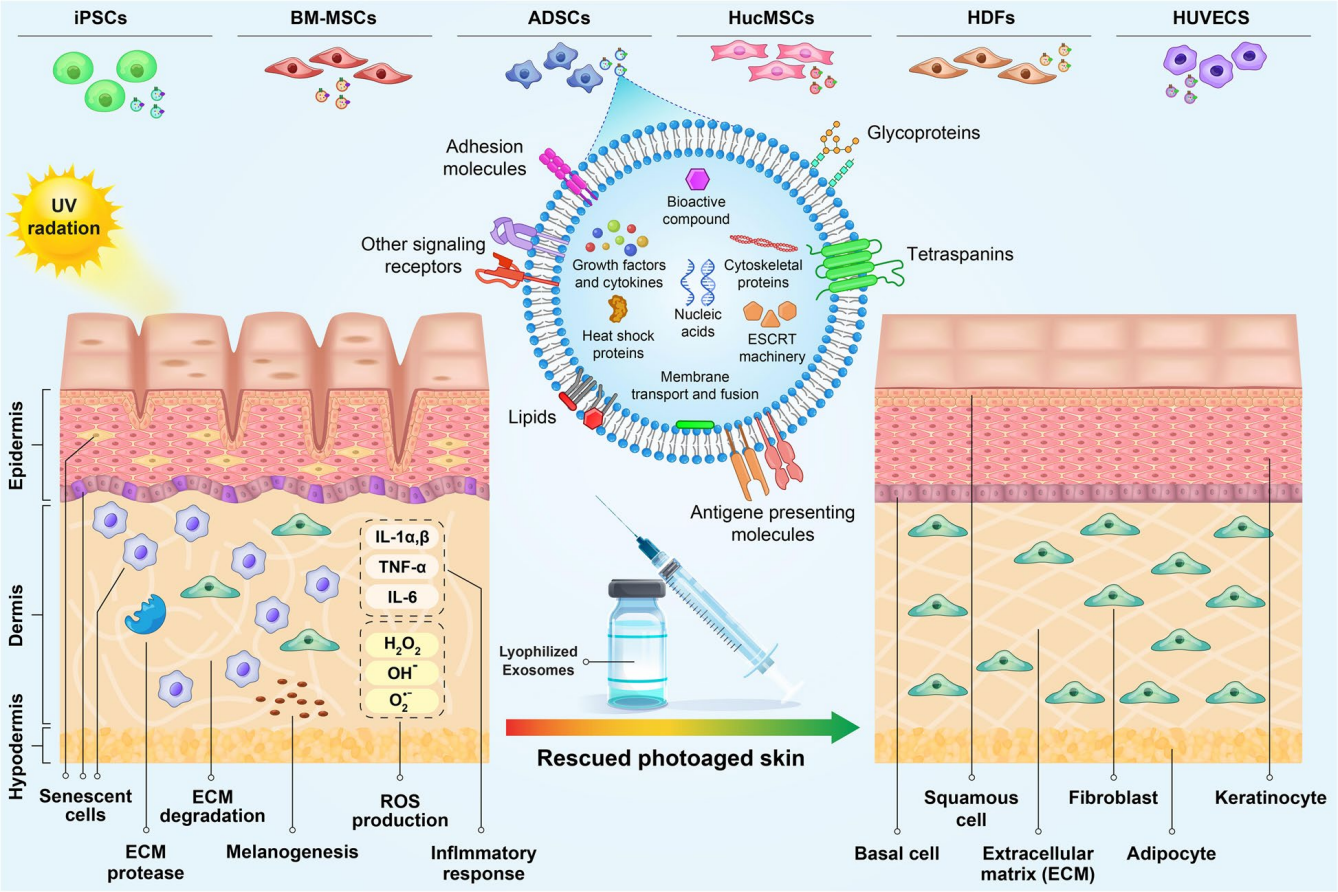
Exosomes derived from different types of stem cells can play an important role in reducing photoaging by entering the target cells and transferring their contents. UV radiation induce generation of reactive oxygen species (ROS), leading to DNA damage, activation of inflammatory pathway, production of matrix metalloproteinases (MMPs) and degradation of collagen fibers. Skin photoaging is characterized by structural change, appearance of wrinkles and pigmentation (Reviewed in [7]). Exosomes derived from stem cells can be served as novel treatment option for skin repair and regeneration. Administering exosomes in the form of lyophilized injection may be one of the effective approaches to repair photo-damaged skin

Exosomes are able to deliver various bioactive compounds into the skin cells, which can effectively delay skin aging and inhibit photoaging signatures. These nanovesicles would be artificially engineered with desired biological molecules [4, 103]. Exosomes can be delivered to skin through various invasive and non-invasive methods. In the non-invasive treatment exosomes are incorporated into topical creams, serums, oils, and masks to cover and protect skin [104]. Exosomes can also be incorporated into bioactive polymeric materials like hydrogel, allowing for sustained release, pH maintenance, and enhanced regenerative potential [105]. Local injection is the invasive type of treatment in which anti-aging molecules are injected into the inner layer of skin to enhance therapeutic effects and overcome skin barrier. Subdermal injection of ADSCs has been demonstrated to be effective in reducing anti-photogaing effects through ECM remodeling and neoelastogenesis (Fig. 2) [106]. Since MSCs-derived exosomes represent biological activity corresponding to these stem cells, similar and even more effective therapeutic outcome is expected in exosome-based therapeutic protocols. Local injection provides more effective skin treatment compared to topical products due to skipping skin barrier [104]. The stability of exosomes is critical both before and after injection. Exosome lyophilization is often used to increase stability and maintain the activity of biological molecules. This method involves in dehydration and drying of exosome under vacuum condition at low temperature, resulting in their longer storage without loss of activity [107]. Systemic treatment is another method previously used to deliver exosomes through intravenous injection. It has been shown that topical application of exosome combined with intravenous injection effectively accelerates non-diabetic wound healing [108]. Exosomes stimulate collagen production in photoaged skin and reduce the appearance of pigmentation [4]. Moreover, photoaging is associated with a greater risk of malignant tumors like melanoma [109]. Thus, treatment of skin photoaging has important clinical significance and exosome-based therapy could be a helpful method not only in cosmetic application but also in skin cancer prevention.

## Conclusion

Photoaging is a prominent manifestation of skin aging characterized by the appearance of mottled pigmentation, fine lines, and wrinkles. The main molecular mechanisms of photoaging are accumulation of reactive oxygen species, cellular senescence, inflammation, and collagen degradation. Targeting these pathways through novel therapeutics is an intriguing area of study in regenerative medicine. Exosomes are able to regulate multiple cellular processes due to their important role in cellular communication. In the last years, exosomes have emerged as a novel therapeutic option for treatment of many diseases. This review aims to summarize the current findings on the roles of exosomes, particularly those derived from stem cells, in the context of skin photoaging. While most studies investigating the use of exosomes in treating skin defects have been conducted at the preclinical level, additional research is needed to evaluate the therapeutic potentials and clinical values of exosomes in the field of skin treatment medicine.

## Data Availability

Not applicable.

## Abbreviations

ROS: Reactive oxygen species
IL: Interleukin
MMP: Matrix metalloproteinase
TLR: Toll-like receptor
MAPK: Mitogen-activated protein kinase
COX-2: Cyclooxygenase-2
TNF-α: Tumor necrosis factor-alpha
NF-κB: Nuclear factor kappa B
IKK: IκB kinase
IκB: Inhibitory kappa B
Nrf2: NF-E2-related factor-2
ARE: Antioxidant response element
SIRT1: Deacetylase silent information regulator 1
PGC-1α: PARγ coactivator-1α
HSF: Human skin fibroblast
MDA: Malondialdehyde
PARP-1: Poly(ADP-ribose) polymerase-1
NET: Neutrophil extracellular traps
ERK: Extracellular signal-regulated kinase
JNK: C-Jun amino-terminal kinase
COL: Collagen
AMPK: AMP-activated protein kinase
UVRAG: UV radiation resistance-associated gene
TSC: Tuberous sclerosis complex
Bach2: BTB and CNC homology 1, basic leucine zipper transcription factor 2
BTB/POZ: Broad complex, tramtrack, bric-a-brac/poxvirus, and zinc finger
HSP: Heat shock protein
DWAT: Dermal white adipose tissue
SWAT: Subcutaneous white adipose tissue
ACC: Acetyl-CoA carboxylase
FAS: Fatty acid synthase
SCD: Stearoyl-CoA desaturase
SREBPs: Sterol regulatory element binding proteins
PPARγ: Peroxisome proliferator-activated receptor
